# DUFfers can be useful—some even save energy! DUF581-9 negatively regulates the key energy sensor SnRK1

**DOI:** 10.1093/plphys/kiad637

**Published:** 2023-12-01

**Authors:** Manuel González-Fuente

**Affiliations:** Assistant Features Editor, Plant Physiology, American Society of Plant Biologists; Faculty of Biology & Biotechnology, Ruhr-University Bochum, Bochum 44801, Germany

Resources are limited. We all are aware of this problem, especially when those unforeseen expenses come and force us to spend part of our monthly income on stuff we initially did not intend to buy. Plants are no different. Their resources are equally limited. Therefore, under unfavorable conditions, such as pathogen attacks or extreme temperatures, plants relocate some of the resources initially intended for growth to counteract these challenges. This trade-off is tightly regulated to ensure an optimal exploitation of resources while maintaining the ability to respond quickly and efficiently to unfavorable conditions. In plants, SUCROSE NON-FERMETING RELATED KINASE 1 (SnRK1) senses energy deficits caused by adverse situations and coordinates the cellular responses to adapt (Baena-González et al. 2007). Whereas the downstream targets of SnRK1 that execute these responses have been extensively studied, the mechanisms that regulate its upstream activation have received less attention (Broeckx et al. 2016). One of the known activators of SnRK1 is GEMINIVIRUS REP INTERACTING KINASE 2 (GRIK2), which functions in the context of viral infection and young tissue development (Shen et al. 2009). It was proposed that, contrary to its animal and yeast counterparts, SnRK1 is not specifically activated upon energy deficit but is rather constitutively active but repressed under energy-sufficient conditions (Ramon et al. 2019). Indeed, several molecules are known to inhibit SnRK1 activity (Broeckx et al. 2016), although the exact molecular mechanisms remain elusive to date.

In this issue of *Plant Physiology*, Bortlik et al. (2023) characterize a novel negative regulator of SnRK1 that binds directly to its catalytic subunit preventing its upstream activation (Fig. 1). In a previous study, the authors showed that proteins with Domain of Unknown Function (DUF) 581 interact with SnRK1 (Nietzsche et al. 2014). To study the biological relevance of these interactions, they focused on DUF581-9, the smallest member of the DUF581 protein family. The authors first observed that *DUF581-9* gene expression is repressed during starvation and induced upon recovery, conditions associated with activation and inactivation of SnRK1, respectively. Moreover, the expression of SnRK1-mediated response marker genes is enhanced in a *duf581-9* mutant and reduced in *DUF581-9* overexpressing lines. This deregulation translates to *DUF581-9* overexpressing lines being strongly affected in their carbohydrate balance, what ultimately leads to coping less well with starvation. Altogether, this shows the implication of DUF581-9 in SnRK1-dependendent responses.

**Figure.**
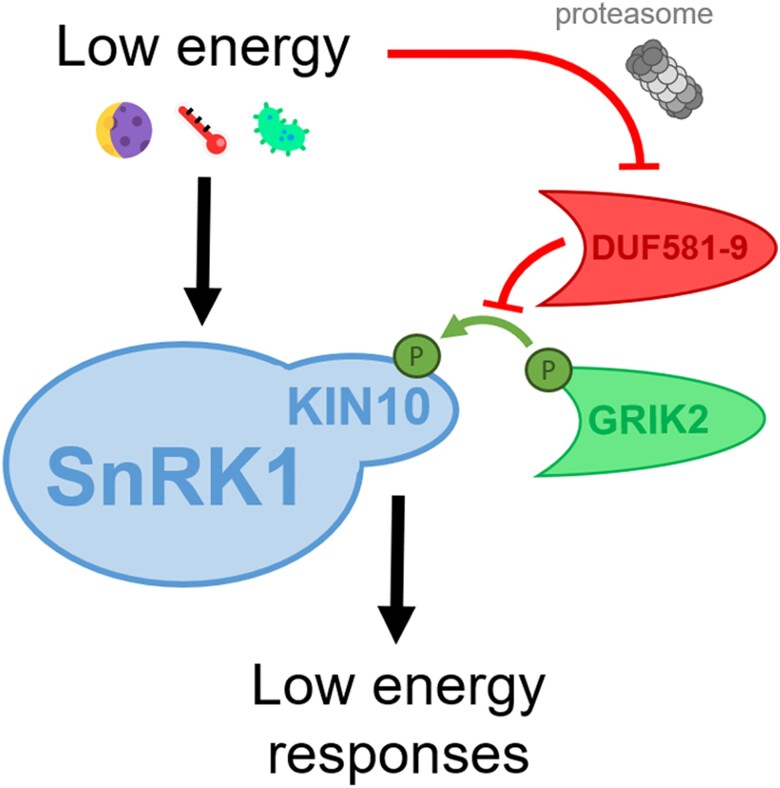
DUF581-9 prevents GRIK2-mediated activation of SnRK1. SnRK1 senses energy deficits (e.g. darkness, extreme temperature, or pathogen attack) and triggers the adequate responses to adapt. SnRK1 activation relies on the phosphorylation of its catalytic subunit, KIN10, by the upstream kinase GRIK2. Under energy-sufficient conditions, SnRK1 activity is repressed by the negative regulator DUF581-9, which sterically prevents the association and thus phosphorylation of KIN10 by GRIK2. Under low-energy conditions, SnRK1 is derepressed by proteasomal degradation of DUF581-9.

To unveil the molecular mechanism behind this DUF581-9–mediated modulation of SnRK1 activity, the authors confirmed the previously reported direct interaction of DUF581-9 with the catalytic subunit of SnRK1, KIN10 (Nietzsche et al. 2014), and reported that this interaction leads to inhibition of SnRK1 activity. Concretely, they revealed that DUF581-9 inhibited KIN10 kinase activity in vitro in a dose-dependent manner and only if DUF581-9 was included at the same time as the upstream activating kinase, GRIK2. As the exact KIN10 region activated/phosphorylated by GRIK2 is known (Shen et al. 2009), the authors observed that the presence of DUF581-9 specifically prevented the phosphorylation of KIN10 at this particular region. Altogether, these results suggest that DUF581-9 inhibits SnRK1 activity by sterically impeding the association of KIN10 with its activating upstream kinase GRIK2.

This work describes a novel and detailed mechanism of inactivation of SnRK1 activity. This could serve as one of the possible mechanisms explaining the proposed constitutive repression of SnRK1 activity in energy-sufficient conditions (Ramon et al. 2019). However, the work raises many questions: How does DUF581-9 behave when SnRK1 needs to be activated? How do energy deficits counteract the negative impact of DUF581-9 on SnRK1? As previously mentioned, the authors initially observed reduced *DUF581-9* gene expression under starvation, but considering the speed of SnRK1 responses, it is likely that additional and faster mechanisms regulate DUF581-9 negative impact on SnRK1 activity. A simple hypothesis would be that preexisting DUF581-9 proteins are degraded to derepress SnRK1, which is in agreement with the observed proteasomal degradation of ectopic DUF581-9 under starvation.

In summary, in this study, Bortlik et al. (2023) describe in detail an additional level of regulation of SnRK1 activity through a novel negative regulator, DUF581-9, that physically impedes KIN10 phosphorylation by the activating kinase GRIK2 in favorable conditions (Fig.). Nevertheless, under starvation, DUF581-9 is partially degraded via the proteasome to derepress SnRK1 activity, thus allowing to execute the required low energy responses. Because proteasomal inhibition recovered DUF581-9 protein levels only partially, it is possible that additional proteolytic pathways, such as autophagy (Raffeiner et al. 2023), are involved in SnRK1 depression as recently reported (Yang et al. 2023). Given the high context-specificity of the complex regulation of SnRK1 activity, this work opens many new questions. For instance, the authors had previously reported that other DUF281-containint proteins also interact with KIN10 (Nietzsche et al. 2014). Do these other interactors also negatively regulate SnRK1 activity in other tissues and/or under other conditions? Interestingly, *GRIK2* and *DUF581-9* gene expression patterns are very similar, being mostly expressed in young developing tissues (Shen et al. 2009; Bortlik et al. 2023). It is thus tempting to speculate that both positive and negative regulators of SnRK1 activity might be tightly interconnected. Whether this kind of tight interplay occurs with other positive and negative regulators of SnRK1 in different tissues or under different circumstances remains to be unveiled. *GRIK2*, as its name suggests (*GEMINIVIRUS REP INTERACTING KINASE 2*), is expressed in virus-infected tissues as well (Shen et al. 2009). Whether *DUF581-9* is also expressed in this condition has not been studied so far, although it would clarify if these 2 interactors are indeed so intrinsically interconnected or if the virus is able to selectively decouple their actions and activate SnRK1 to its own benefit.

Given the biological relevance of SnRK1, this work contributes to the overall understanding of the complex and multilayered regulation of a key energetic sensor (Baena-González et al. 2007). As such, this knowledge will not only contribute to better understand the maintenance of the cellular carbon balance and overall energetic homeostasis, but given the potential of these pathways in agronomy (Paul et al. 2020), it will also serve to develop in the future novel crop varieties with higher resilience and yield.

## Dive Curated Terms

The following phenotypic, genotypic, and functional terms are of significance to the work described in this paper:

DUF581-9 Gramene: AT2G44670
DUF581-9 Araport: AT2G44670

KIN10 Gramene: AT3G01090
KIN10 Araport: AT3G01090

GRIK2 Gramene: AT5G60550
GRIK2 Araport: AT5G60550
